# Supplementary material for: Chromosome-level genome assemblies reveal genome evolution of an invasive plant Phragmites australis
Source: Commun Biol. 2024 Aug 17;7:1007. doi: 10.1038/s42003-024-06660-1 (PMC11330502; doi:10.1038/s42003-024-06660-1)
Supplement: Supplementary file 2 — Supplementary Material [file 42003_2024_6660_MOESM2_ESM.pdf]

Supplementary Materials for

Chromosome-level genome assemblies reveal genome evolution of an invasive plant *Phragmites australis*

Cui Wang *et al*.

*Corresponding author. Email: whguo@sdu.edu.cn, jarkko@ntu.edu.sg

This PDF file includes:
Figure S1-S11

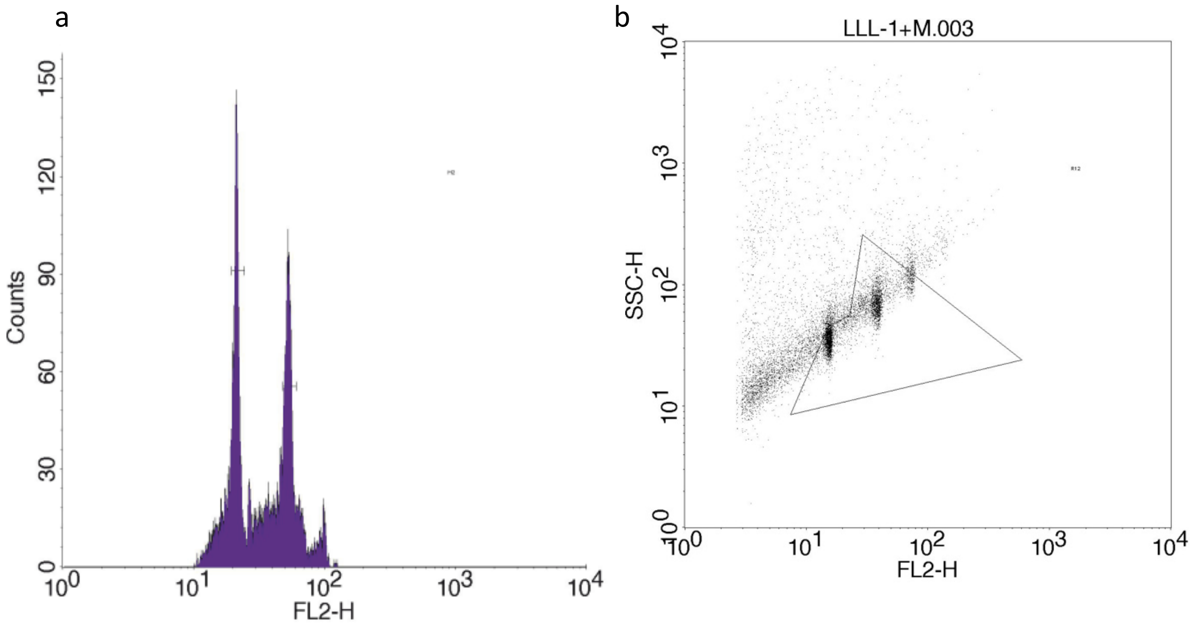

Figure S1. a. The DNA content per cell estimated using flow cytometry suggests a genome size of 920Mb for *P. australis* (left peak). Maize (*Zea mays*) was used as a control (right peak). b. Forward and side scatter gating strategy used for the flow cytometry measurement.

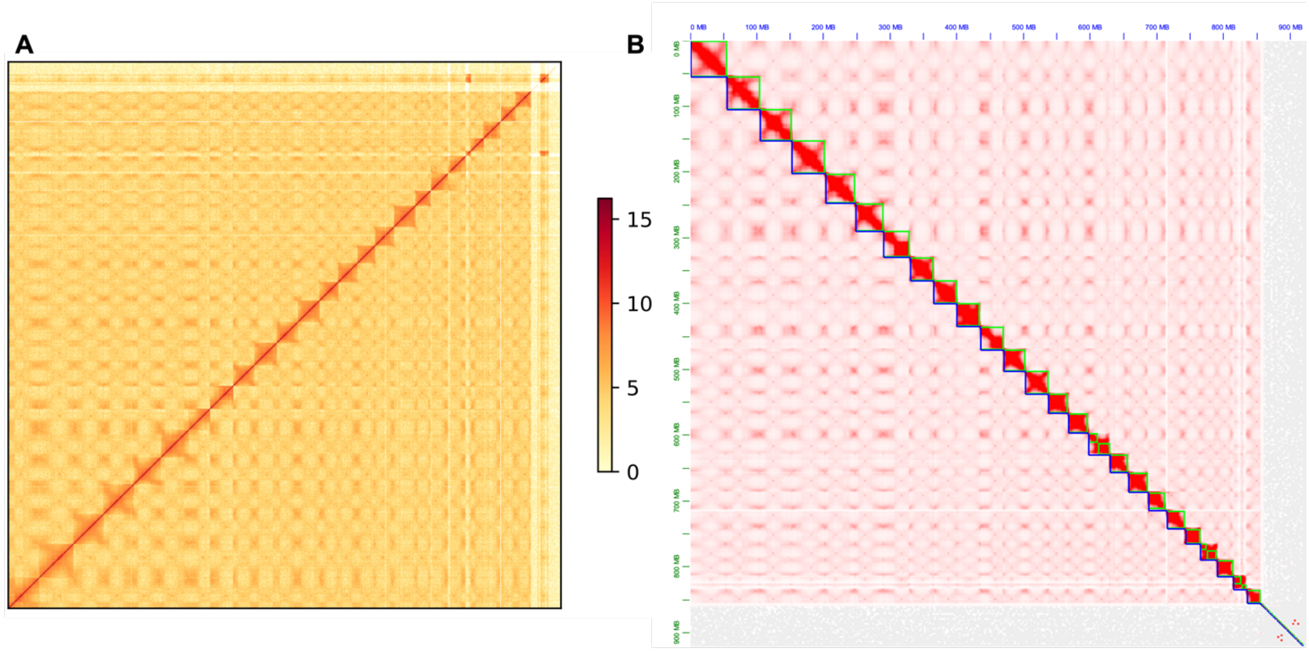

Figure S2. Hi-C contact heat maps generated by aligning Hi-C short read sequences against the draft assembly. **A.** The initial draft was assembled from with Pacbio HiFi reads and then scaffolded into 25 pseudomolecules using Hi-C contacts using 100x coverage data and the ALLHiC pipeline. The heatmap displays the contiguous blocks (pseudomolecules) after scaffolding the draft assembly into 25 pseudochromosomes. One small block in the top right corner, showing substantial contacts with the fourth block from the top was manually corrected. **B.** After manual correction using Juicebox and the 3d-dna pipeline, the assembly errors were resolved.

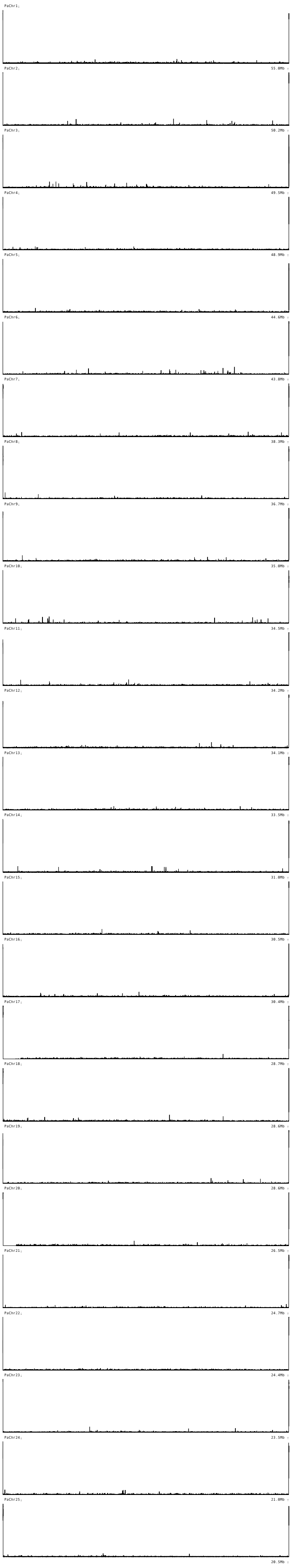
PaChr1
PaChr2
55.0Mb
PaChr3
50.2Mb
PaChr4
49.5Mb
PaChr5
48.9Mb
PaChr6
44.6Mb
PaChr7
43.8Mb
PaChr8
38.3Mb
PaChr9
36.7Mb
PaChr10
35.0Mb
PaChr11
34.5Mb
PaChr12
34.2Mb
PaChr13
34.1Mb
PaChr14
33.5Mb
PaChr15
31.0Mb
PaChr16
30.5Mb
PaChr17
30.4Mb
PaChr18
28.7Mb
PaChr19
28.6Mb
PaChr20
28.6Mb
PaChr21
26.5Mb
PaChr22
24.7Mb
PaChr23
24.4Mb
PaChr24
23.5Mb
PaChr25
21.0Mb
20.5Mb

Figure S3. Chromosomal regions with telomere repeat counts detected using a sliding window size of 10,000 bp. Telomeres were detected at both ends of all of the 25 pseudochromosomes.

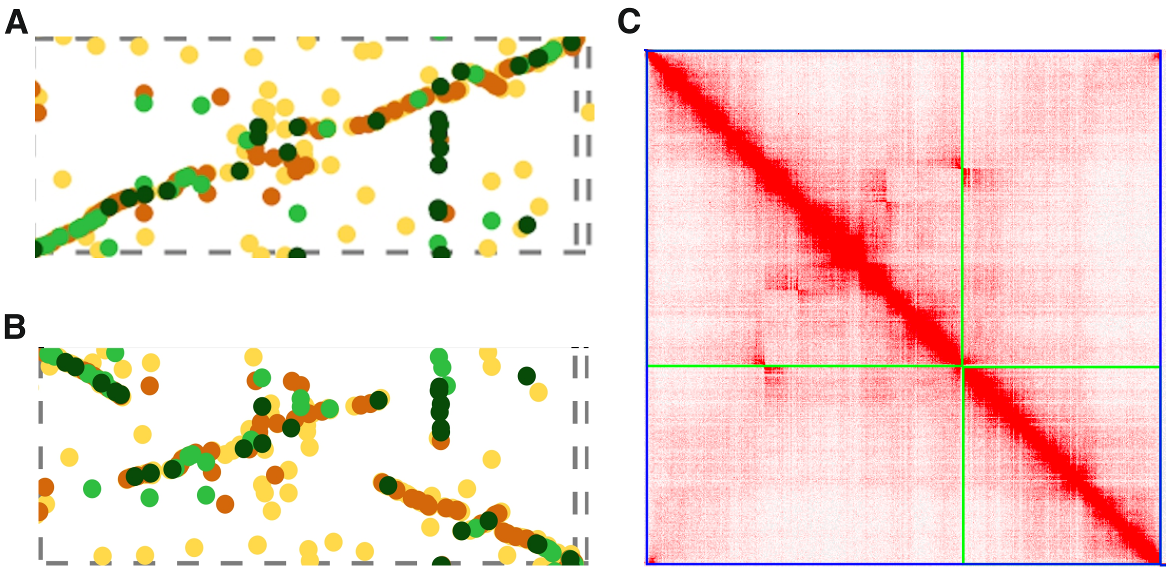

Figure S4. A large haplotypic inversion was detected on Chromosome 22. The two haplotig assemblies from Hifiasm synteny analysis between the two haplotypes and rice (**A** and **B**) indicated an inversion in one haplotype (**B**). This inversion is also evident from the Hi-C contact heatmap (**C**).

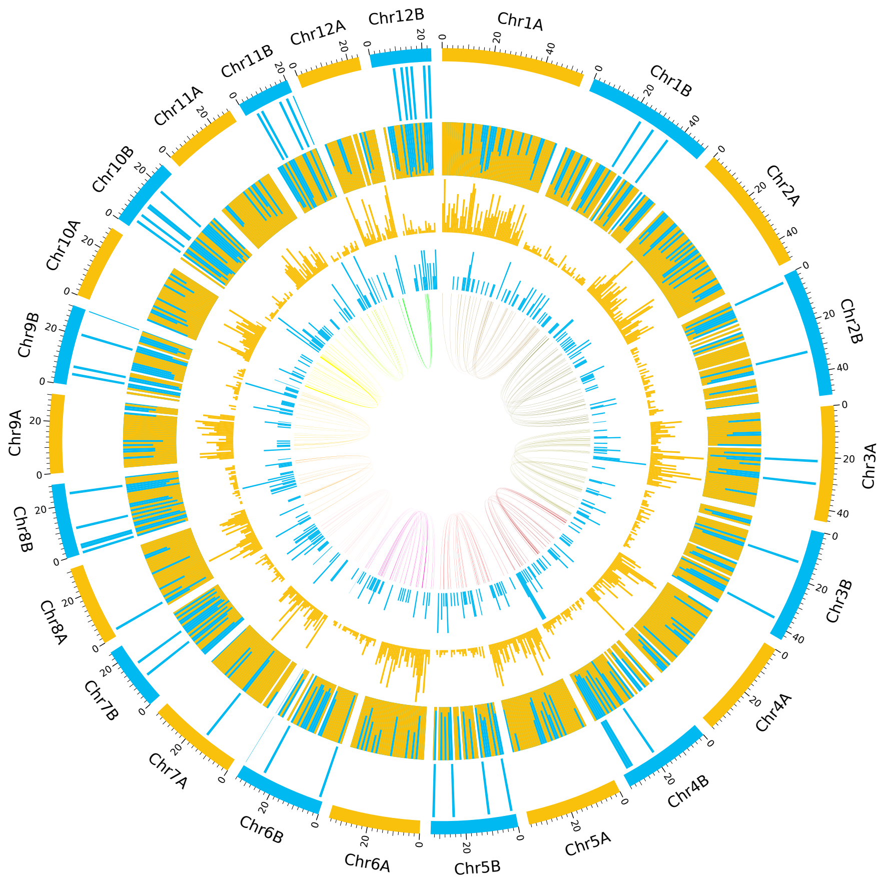

Figure S5. The homoeologous chromosome pairs for the two subgenomes revealed by Subphaser. Chromosomal characteristics from outer to inner circles indicates (1-6): (1) subgenome assignments based on k-means algorithm; (2) significant enrichment of subgenome-specific k-mers – the same color as the subgenome indicates significant enrichment for those subgenome-specific k-mers; white areas are not significantly enriched; (3) normalized proportion (relative) of subgenome-specific k-mers; (4 – 5) count (absolute) of each subgenome-specific k-mer set; (6) homoeologous blocks.

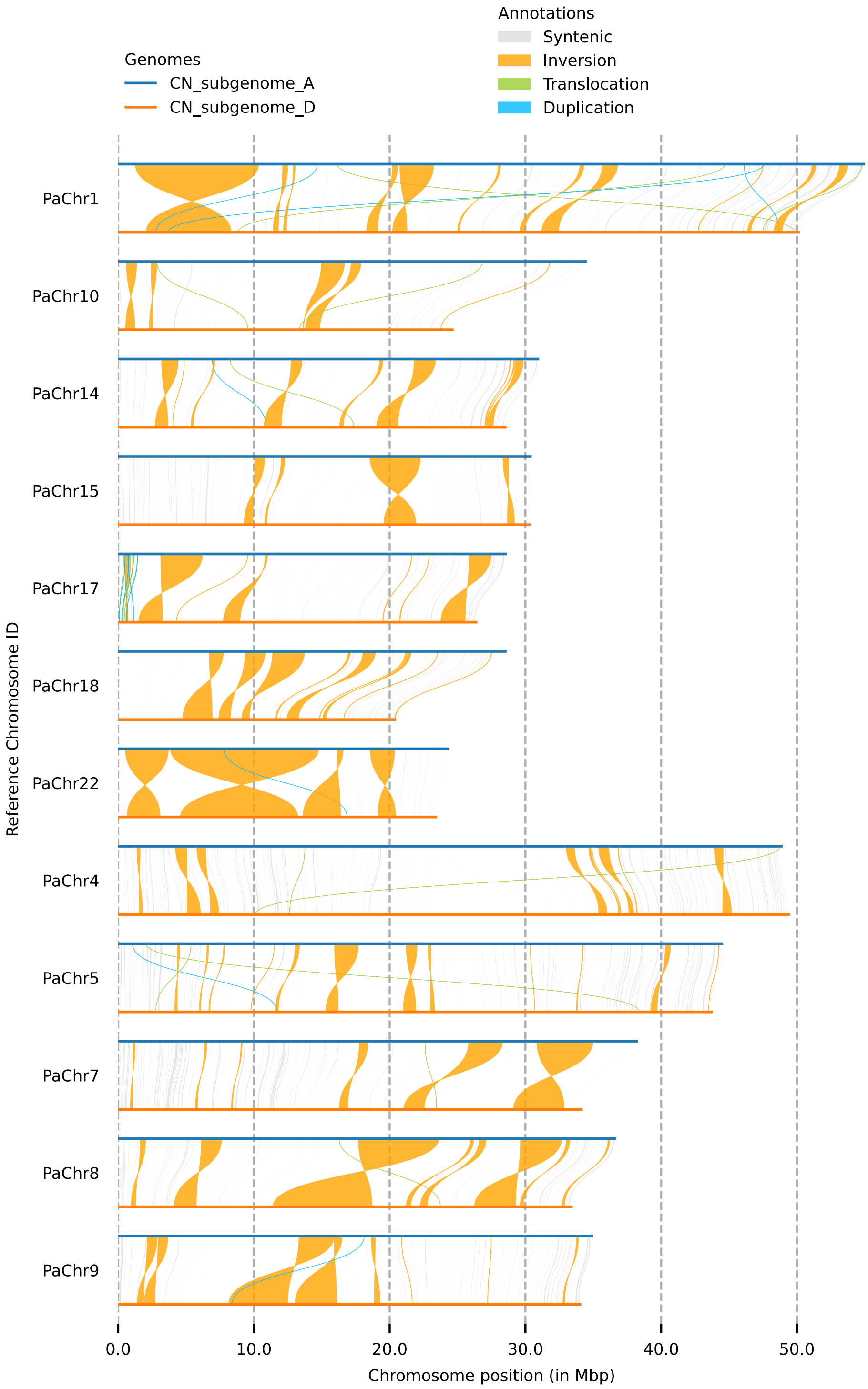

Figure S6. Genome rearrangements between the two subgenomes of *P. australis*. Large inversions were detected in several homoeologous chromosome pairs.

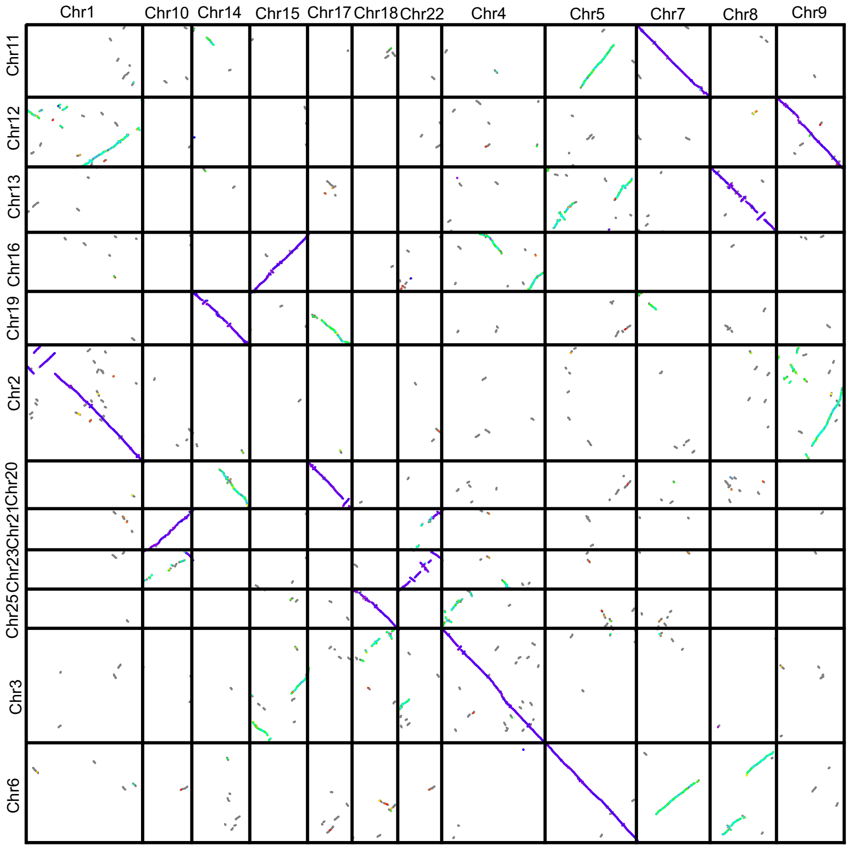

Figure S7. Syntenic dotplot of the two subgenomes of *P. australis* aligned against each other. Colors correspond to the number of synonymous mutations (Ks) between syntelogs, with purple syntenic regions originating from the common reed allopolyploid event (more recent) and green, yellow and red correspond to synteny blocks originating from the earlier whole genome duplication events.

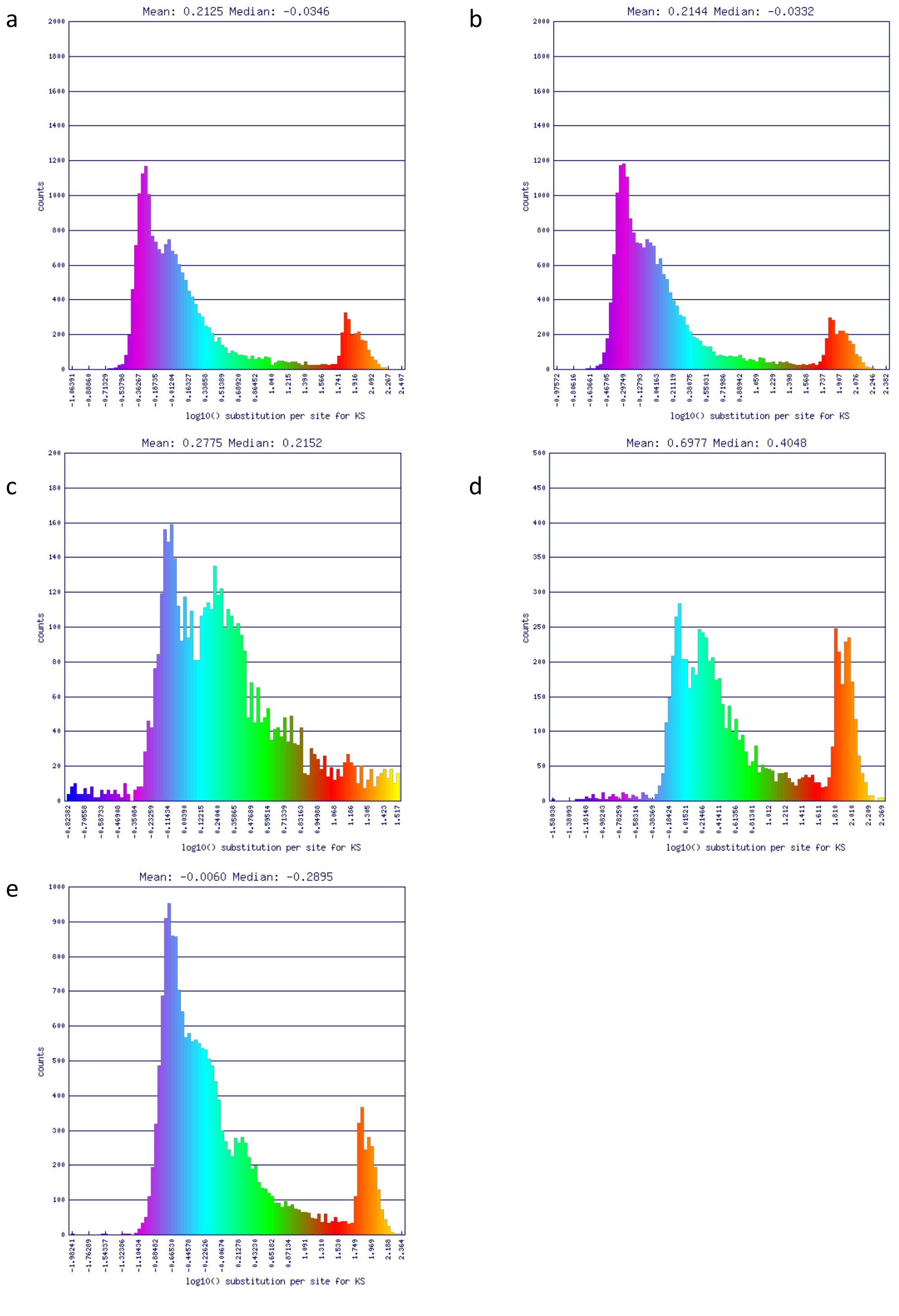

Figure S8. Synonymous substitution (Ks) histograms for the syntenic genes between subgenomes of the reference *P. australis* and rice (*Oryza sativa*), as well as self-self alignments of subgenomes. The panels show histograms of Ks values transformed with log10 when aligning **(a)** subgenome A to rice genome, **(b)** subgenome D to rice genome, **(c)** subgenome A to itself, **(d)** subgenome D to itself, **(e)** the two subgenomes against each other. Ks value on the x-axis was. For all the figures, the orange peaks on the right with the transformed Ks values higher than 1.6 are noise due to mis-called syntenic pairs or wrong prediction of gene models. The time of whole genome duplication (WGD) events in subgenome A and D can be inferred from the self-self alignments from each subgenome (**c, d**) with two peak values higher than -0.13, suggests all the WGD events are predating the

divergence date with rice which shows a most recent peak at around -0.29. Divergence time between the subgenomes can be inferred from the most recent peak of E, at around -0.63.

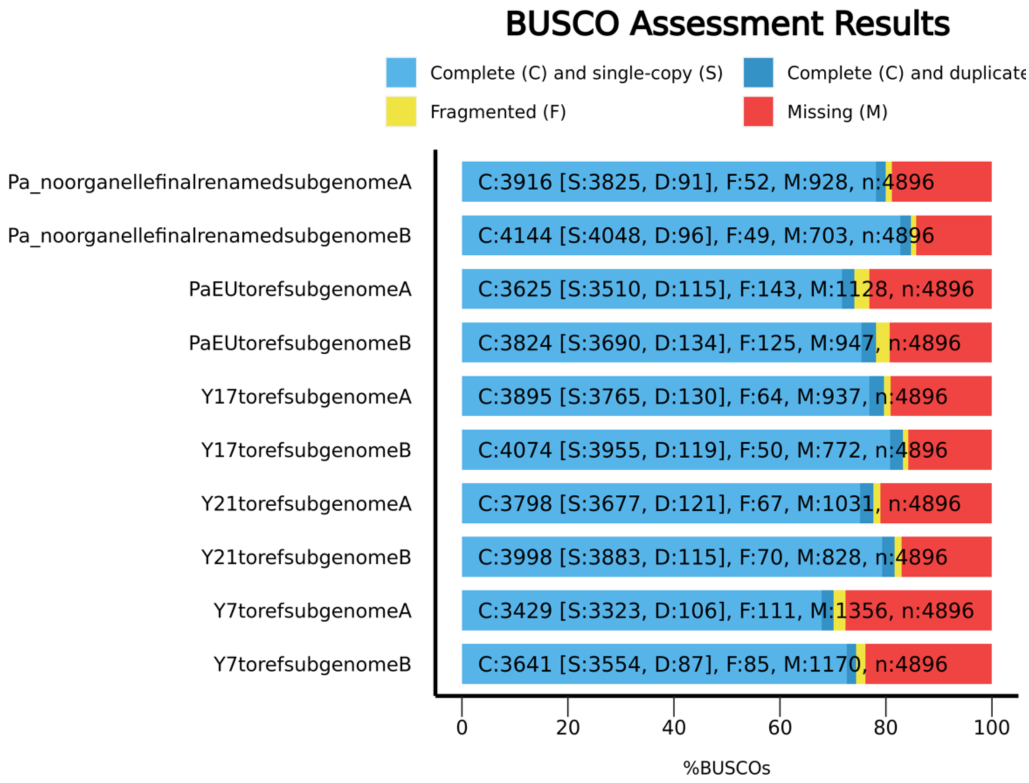

Figure S9. BUSCO assessment for all the subgenomes of the five-representative lineages. From the top to bottom, the first two bar plots show the BUSCO qualities for the subgenome A and D for Chinese reference individual genome assembly. Draft genomes of the other four genomes were scaffolded into chromosome-level using their homology to the reference genome, subgenomes were phased in this process. From the third bar plot to the bottom, the chart shows BUSCO scores for the EU invasive lineage subgenomes A, and D, North American native lineage (Y17, USnat) subgenomes A and D, Mediterranean lineage (Y21, Med) subgenomes A and D, as well as Gulf Coast Land type (Y7, USland) subgenomes A and D.

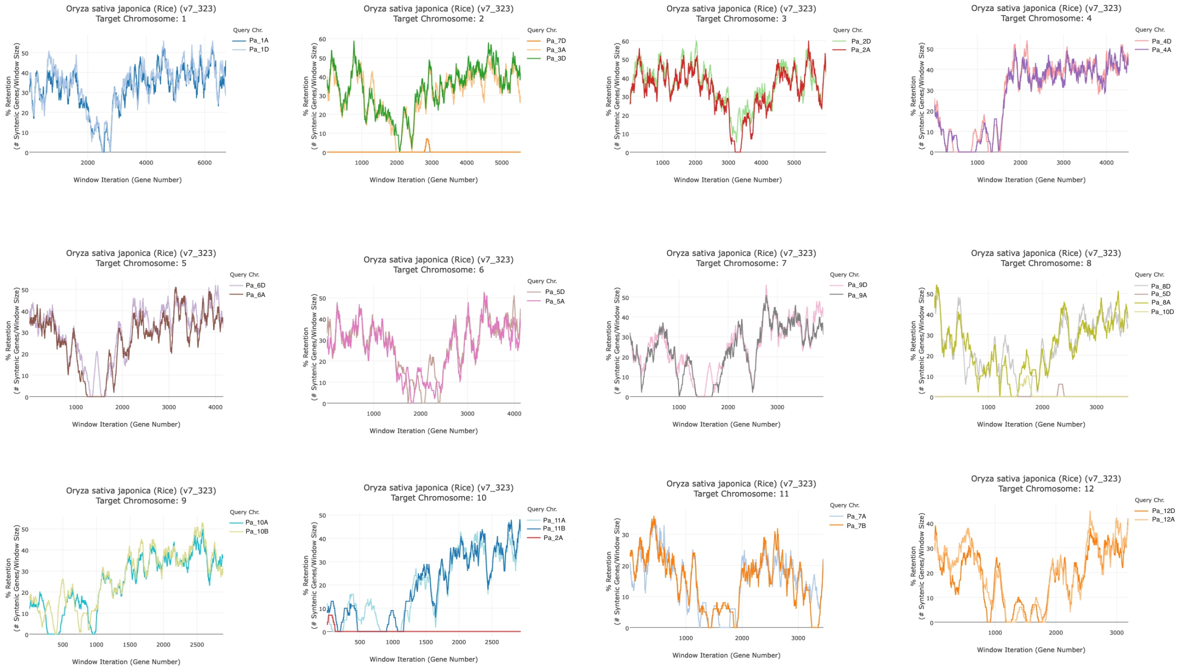

Figure S10. Fractionation biases of subgenome D vs subgenome A in *P. australis* reference, when aligned against rice. The charts show the number of syntenic genes retained in sliding genomic windows of 100 genes along the homoeologous chromosome pairs. The gene order on x axis follows the chromosome ordering of *O. sativa japonica*. The overall fractionation is a result from the divergence between *O. sativa* and the common ancestor of *P. australis* progenitors, while the differences between the subgenome graphs suggest biased gene retention.

a

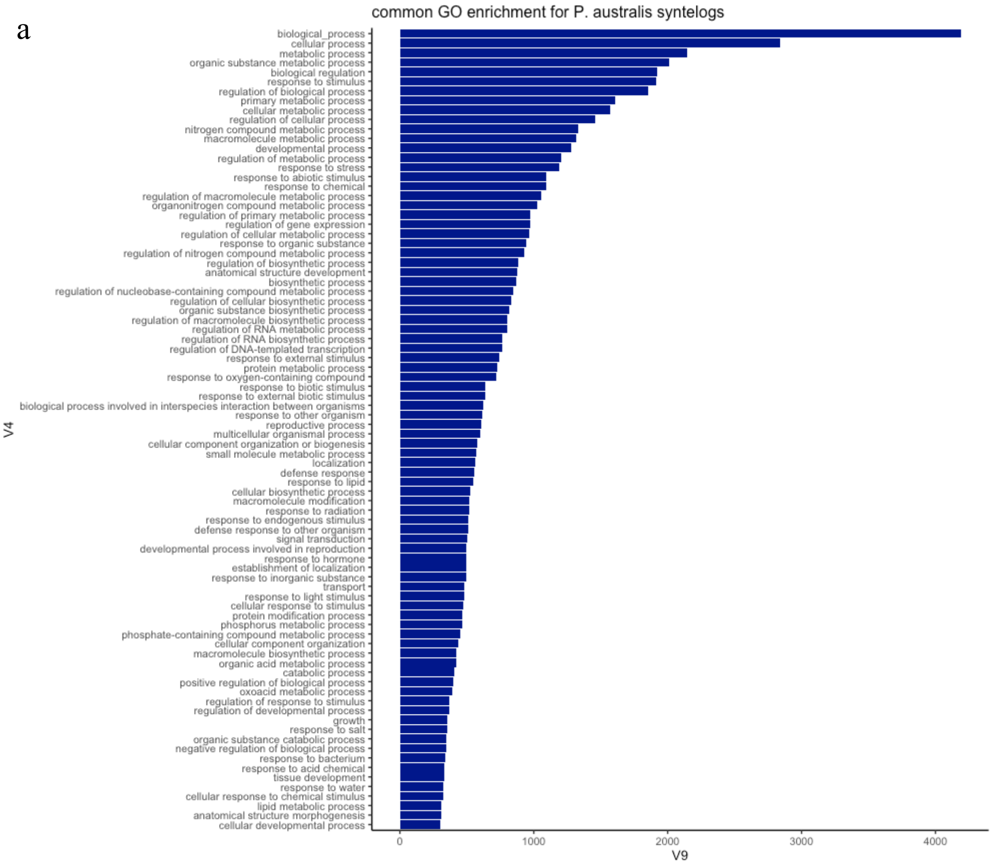

common GO enrichment for P. australis syntelogs
biological_process
cellular process
metabolic process
organic substance metabolic process
biological regulation
response to stimulus
regulation of biological process
primary metabolic process
cellular metabolic process
regulation of cellular process
nitrogen compound metabolic process
macromolecule metabolic process
developmental process
regulation of metabolic process
response to stress
response to abiotic stimulus
response to chemical
regulation of macromolecule metabolic process
organonitrogen compound metabolic process
regulation of primary metabolic process
regulation of gene expression
regulation of cellular metabolic process
response to organic substance
regulation of nitrogen compound metabolic process
regulation of biosynthetic process
anatomical structure development
biosynthetic process
regulation of nucleobase-containing compound metabolic process
regulation of cellular biosynthetic process
organic substance biosynthetic process
regulation of macromolecule biosynthetic process
regulation of RNA metabolic process
regulation of RNA biosynthetic process
regulation of DNA-templated transcription
response to external stimulus
protein metabolic process
response to oxygen-containing compound
response to biotic stimulus
response to external biotic stimulus
biological process involved in interspecies interaction between organisms
response to other organism
reproductive process
multicellular organismal process
cellular component organization or biogenesis
small molecule metabolic process
localization
defense response
response to lipid
cellular biosynthetic process
macromolecule modification
response to radiation
response to endogenous stimulus
defense response to other organism
signal transduction
developmental process involved in reproduction
response to hormone
establishment of localization
response to inorganic substance
transport
response to light stimulus
cellular response to stimulus
protein modification process
phosphorus metabolic process
phosphate-containing compound metabolic process
cellular component organization
macromolecule biosynthetic process
organic acid metabolic process
catabolic process
positive regulation of biological process
oxoacid metabolic process
regulation of response to stimulus
regulation of developmental process
growth
response to salt
organic substance catabolic process
negative regulation of biological process
response to bacterium
response to acid chemical
tissue development
response to water
cellular response to chemical stimulus
lipid metabolic process
anatomical structure morphogenesis
cellular developmental process
V4
0
1000
2000
3000
4000
V9

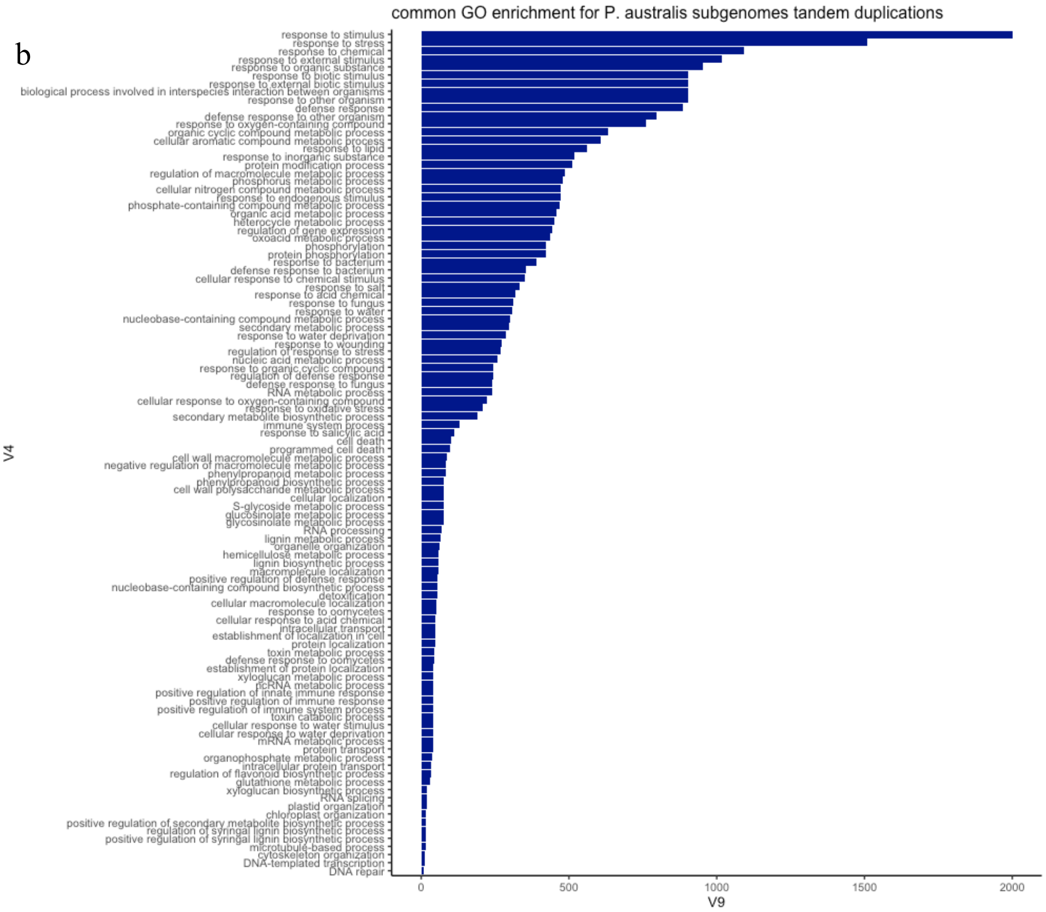

Figure S11. Significantly enriched GO terms shared between subgenome A and subgenome D for the genes originating from **(a)** whole genome duplications and **(b)** tandem duplications. The statistics showed their enrichment in subgenome A.
