# Supplementary material for: Chromosome-level genome assemblies reveal genome evolution of an invasive plant Phragmites australis
Source: Commun Biol. 2024 Aug 17;7:1007. doi: 10.1038/s42003-024-06660-1 (PMC11330502; doi:10.1038/s42003-024-06660-1)
Supplement: Supplementary file 3 — Decscription of Additional Supplementary Files [file 42003_2024_6660_MOESM3_ESM.pdf]

Description of Additional Supplementary Files

File name: Supplementary Data 1

Description: Repeat types of the five *P.australis* genome assemblies.

File name: Supplementary Data 2

Description: Genome assembly and quality statistics for the three contig-level genome assemblies obtained from genome assembly of Illumina sequencing data.

File name: Supplementary Data 3

Description: Results from scaffolding of the draft assemblies to chromosome level when using the reference assembly as template.

File name: Supplementary Data 4

Description: Chromosome sizes (in base pairs, bp) of each of the diffent lineages obtained from scaffoding.

File name: Supplementary Data 5

Description: Gene ontology (GO) enrichment analysis of subgenome A and subgenome B syntelogs. The statistics shows the enrichment results in subgenome A.

File name: Supplementary Data 6

Description: Gene ontology (GO) enrichment analysis of tandemly duplicated genes in subgenomes A and B. The statistics shows the GO enrichment in subgenome A.

File name: Supplementary Data 7

Description: The species used in gene family evolutionary analyses. The table lists the genomes of closely related species of P. australis, their annotation qualities and references to the original publications.

File name: Supplementary Data 8

Description: Gene ontology (GO) enrichment analysis of the genes specific to the PaEU invasive lineage.

File name: Supplementary Data 9

Description: Gene ontology (GO) enrichment analysis of the tandemly duplicated genes in the PaEU invasive lineage.

File name: Supplementary Data 10

Description: Accession numbers of the RNAseq libraries used for genome annotation, the last two columns state whether B chromosome genes were expressed and the numbers of alternatively spliced isoforms detected in B chromosomes.
