# Supplementary material for: Chromosome-level genome assemblies reveal genome evolution of an invasive plant Phragmites australis
Source: Commun Biol. 2024 Aug 17;7:1007. doi: 10.1038/s42003-024-06660-1 (PMC11330502; doi:10.1038/s42003-024-06660-1)
Supplement: Supplementary file 5 — Reporting summary [file 42003_2024_6660_MOESM5_ESM.pdf]

nature portfolio

Corresponding author(s): Cui Wang (cui.wang@helsinki.fi)
Weihua Guo (whguo@sdu.edu.cn)
Jarkko Salojärvi (jarkko@ntu.edu.sg)

Last updated by author(s): Jun 17, 2024

Reporting Summary

Nature Portfolio wishes to improve the reproducibility of the work that we publish. This form provides structure for consistency and transparency in reporting. For further information on Nature Portfolio policies, see our Editorial Policies and the Editorial Policy Checklist.

Statistics

For all statistical analyses, confirm that the following items are present in the figure legend, table legend, main text, or Methods section.

n/a	Confirmed	
☒	☐	The exact sample size (*n*) for each experimental group/condition, given as a discrete number and unit of measurement
☒	☐	A statement on whether measurements were taken from distinct samples or whether the same sample was measured repeatedly
☐	☒	The statistical test(s) used AND whether they are one- or two-sided<br>*Only common tests should be described solely by name; describe more complex techniques in the Methods section.*
☒	☐	A description of all covariates tested
☐	☒	A description of any assumptions or corrections, such as tests of normality and adjustment for multiple comparisons
☒	☐	A full description of the statistical parameters including central tendency (e.g. means) or other basic estimates (e.g. regression coefficient) AND variation (e.g. standard deviation) or associated estimates of uncertainty (e.g. confidence intervals)
☐	☒	For null hypothesis testing, the test statistic (e.g. *F*, *t*, *r*) with confidence intervals, effect sizes, degrees of freedom and *P* value noted<br>*Give P values as exact values whenever suitable.*
☒	☐	For Bayesian analysis, information on the choice of priors and Markov chain Monte Carlo settings
☒	☐	For hierarchical and complex designs, identification of the appropriate level for tests and full reporting of outcomes
☒	☐	Estimates of effect sizes (e.g. Cohen's *d*, Pearson's *r*), indicating how they were calculated

Our web collection on statistics for biologists contains articles on many of the points above.

Software and code

Policy information about availability of computer code

Data collection	N.A.
Data analysis	The scripts used for the construction of genome assembly and genome annotation, as well as data analysis in this article could be found from https://github.com/smallfishcui/Phrag_Genome. We used the following softwares for data analysis: PMAT v1.5.3; Flye v2.9.3; GetOrganelle v1.7.7.0; Bandage v0.9.0; Hifiasm v0.19.8-r603; ALLHiC v0.9.13; juicer v1.6; juicebox v2.20.00; 3D-DNA Phasing branch 201008; TGS-GapCloser v1.2.1; KmerGenie v1.7048; FastQC v0.12.0; MaSuRCA v4.0.9; purge_haplotigs v1.0.4; Quast v5.2.0; RagTag v2.1.0; LTR_retriever v2.9.8; RepeatMasker v4.1.6; tRNAscan-SE v2.0.12; Infernal v1.1.5; GeneMark-ES; BRAKER v3.0.7; PASApipeline v2.5.3; AUGUSTUS v3.5.0; GeMoMa v1.9; STAR aligner v2.7.11b; Trinity v2.15.1; stringtie v2.2.3; TransDecoder v5.7.1; EVidenceModeler v2.1.0; DIAMOND v2.1.8; Benchmarking Universal Single Copy Orthologs v5.1.2; GeneOverlap v1.40.0; repeatR v0.1-alpha; D-GENIES; SubPhaser v1.2; NgenomeSyn v1.41; Syri v1.7.0; goatools v1.4.11; OrthoFinder v2.5.5; cogeqc v1.8.0; karyoploteR v1.8.4; RaxML v8.2.12; Astral-III v5.7.1; PAML v4.9e; bwa v0.7.17; PSMC v0.6.5; PiNSiR; samtools v1.16.1; bcftools v1.16; ANGSD v 0.940-stable; SnpEff v5.2b; R v4.2.

For manuscripts utilizing custom algorithms or software that are central to the research but not yet described in published literature, software must be made available to editors and reviewers. We strongly encourage code deposition in a community repository (e.g. GitHub). See the Nature Portfolio guidelines for submitting code & software for further information.

Data

Policy information about availability of data

All manuscripts must include a data availability statement. This statement should provide the following information, where applicable:

- Accession codes, unique identifiers, or web links for publicly available datasets
- A description of any restrictions on data availability
- For clinical datasets or third party data, please ensure that the statement adheres to our policy

The raw sequencing data are available in NCBI SRA database under the BioProject ID PRJNA849004. This reference genome assembly has been deposited at DDBJ/ENA/GenBank under the accession JBCHVN000000000. The version described in this paper is version JBCHVN010000000. The assemblies of all the accessions and their annotations are available at CoGe (https://genomevolution.org/coge/): the chromosome-level reference CN individual (id 66002), scaffolded North American invasive lineage (id67053), Med lineage (Y21, id67057), USland lineage (Y7, id67058), and USnat lineage (Y17, id67059). The assemblies, the annotations, chloroplast and mitochondrial genome assemblies as well as supplementary data can be downloaded from Figshare: https://figshare.com/projects/Phragmites_genome_evolution/174864.

Research involving human participants, their data, or biological material

Policy information about studies with human participants or human data. See also policy information about sex, gender (identity/presentation), and sexual orientation and race, ethnicity and racism.

Reporting on sex and gender	N.A.
Reporting on race, ethnicity, or other socially relevant groupings	N.A.
Population characteristics	N.A.
Recruitment	N.A.
Ethics oversight	N.A.

Note that full information on the approval of the study protocol must also be provided in the manuscript.

Field-specific reporting

Please select the one below that is the best fit for your research. If you are not sure, read the appropriate sections before making your selection.

☐ Life sciences ☐ Behavioural & social sciences ☒ Ecological, evolutionary & environmental sciences

For a reference copy of the document with all sections, see nature.com/documents/nr-reporting-summary-flat.pdf

Ecological, evolutionary & environmental sciences study design

All studies must disclose on these points even when the disclosure is negative.

Study description	Chromosome level genome assembly of common reed and comparative genomics among genetic lineages
Research sample	Four samples of common reed, representing four genetic lineages.
Sampling strategy	Leaves of these four samples were taken for DNA extraction. We obtained Pacbio HiFi reads and HiC sequencing data for one sample to get the chromosome level genome assembly. Whole genome sequencing using illumina sequencing method were performed on the other three samples to get the whole genome sequences. One sample is enough for genome assembling.
Data collection	Lele. Liu and Cui Wang collected the leaves and performed DNA extractions. Biological companies performed DNA sequencing and provided data to Cui Wang. The DNA genome assembly of a individual representing North American invasive lineage was obtained from a published research "Novel genome characteristics contribute to the invasiveness of Phragmites australis (common reed)". The ID in Coge database is "59768".
Timing and spatial scale	The timing doesn't affect the genome content of the samples. Samples were collected between 2020-2021 from a common garden in China. The spatial scale is global.
Data exclusions	No data was excluded.
Reproducibility	Data analysis were done using different sets of parameters and different softwares. The results support each other well. Several trials were performed to assemble the chromosome level genome assembly.

Randomization	Random samples were selected from different geographic origins to represent different lineages.
Blinding	N.A.

Did the study involve field work? ☐ Yes ☒ No

Reporting for specific materials, systems and methods

We require information from authors about some types of materials, experimental systems and methods used in many studies. Here, indicate whether each material, system or method listed is relevant to your study. If you are not sure if a list item applies to your research, read the appropriate section before selecting a response.

Materials & experimental systems

n/a	Involved in the study	
☒	☐	Antibodies
☒	☐	Eukaryotic cell lines
☒	☐	Palaeontology and archaeology
☒	☐	Animals and other organisms
☒	☐	Clinical data
☒	☐	Dual use research of concern
☐	☒	Plants

Methods

n/a	Involved in the study	
☒	☐	ChIP-seq
☐	☒	Flow cytometry
☒	☐	MRI-based neuroimaging

Dual use research of concern

Policy information about dual use research of concern

Hazards

Could the accidental, deliberate or reckless misuse of agents or technologies generated in the work, or the application of information presented in the manuscript, pose a threat to:

No	Yes	
☒	☐	Public health
☒	☐	National security
☒	☐	Crops and/or livestock
☒	☐	Ecosystems
☒	☐	Any other significant area

Experiments of concern

Does the work involve any of these experiments of concern:

No	Yes	
☒	☐	Demonstrate how to render a vaccine ineffective
☒	☐	Confer resistance to therapeutically useful antibiotics or antiviral agents
☒	☐	Enhance the virulence of a pathogen or render a nonpathogen virulent
☒	☐	Increase transmissibility of a pathogen
☒	☐	Alter the host range of a pathogen
☒	☐	Enable evasion of diagnostic/detection modalities
☒	☐	Enable the weaponization of a biological agent or toxin
☒	☐	Any other potentially harmful combination of experiments and agents

Plants

Seed stocks

The common reed samples were sampled from a common garden in China. The original location of these samples are China, United States, and Lybia. The sampling time was March, 2021. Leaves of the plant were taken for DNA extraction.

Novel plant genotypes

All the samples were wild types.

Authentication

The samples used in the current study have been used in previous genetic studies, and the current study itself is authenticating the phylogenetic relationship of the species.

Flow Cytometry

Plots

Confirm that:

- [ ] The axis labels state the marker and fluorochrome used (e.g. CD4-FITC).
- [ ] The axis scales are clearly visible. Include numbers along axes only for bottom left plot of group (a 'group' is an analysis of identical markers).
- [ ] All plots are contour plots with outliers or pseudocolor plots.
- [x] A numerical value for number of cells or percentage (with statistics) is provided.

Methodology

Sample preparation

plant tissue was initially digested with pre-cooled MG solution (45 mM $MgCl2·6H2O$, 20 mM MOPS, 30 mM Sodium citrate, 1% (W/V) PVP40， 0.2% (v/v) Tritonx-100,10mM Na2EDTA, 20μL/mL β-mercaptoethanol, pH 7.5). The tissue was then broken apart thoroughly using sharp blade and placed still on ice for 10 min, followed by filtration using 40μm diameter mesh to obtain the cell suspension. Add 50 μg/mL propidium iodide and RNAase, then place 16 the cell suspension on ice in dark for staining.

Instrument

BD FACScalibur

Software

Modifit3.0

Cell population abundance

Zea mays B73 was used as calibrations, and mixed with the sample to be tested. 10000 cells were collected.

Gating strategy

Events are detected by FL2 channel in logarithmic mode FL2-H. The coefficient of variation (CV) is less than 5%. Three replicates were done.

- [x] Tick this box to confirm that a figure exemplifying the gating strategy is provided in the Supplementary Information.
